# Genetic diversity and differentiation patterns in *Micromeria* from the Canary Islands are congruent with multiple colonization dynamics and the establishment of species syngameons

**DOI:** 10.1186/s12862-017-1031-y

**Published:** 2017-08-22

**Authors:** M. Curto, P. Puppo, S. Kratschmer, H. Meimberg

**Affiliations:** 1 Institute for Integrative Nature Conservation Research, University of Natural Resources and Life Sciences, A-1180 Vienna, Austria; 2 CIBIO, Research Center in Biodiversity and Genetic Resources / InBio Associated Laboratory, University of Porto, Campus Vairão, 4485-661 Vairão, Portugal

**Keywords:** Hybrid swarm, Species syngameon, Genetic diversity, Hybridization, Genetic structure, Oceanic islands

## Abstract

**Background:**

Especially on islands closer to the mainland, such as the Canary Islands, different lineages that originated by multiple colonization events could have merged by hybridization, which then could have promoted radiation events (Herben et al., J Ecol 93: 572–575, 2005; Saunders and Gibson, J Ecol 93: 649–652, 2005; Caujapé-Castells, Jesters, red queens, boomerangs and surfers: a molecular outlook on the diversity of the Canarian endemic flora, 2011). This is an alternative to the scenario where evolution is mostly driven by drift (Silvertown, J Ecol 92: 168–173, 2004; Silvertown et al., J Ecol 93: 653–657, 2005). In the former case hybridization should be reflected in the genetic structure and diversity patterns of island species. In the present work we investigate *Micromeria* from the Canary Islands by extensively studying their phylogeographic pattern based on 15 microsatellite loci and 945 samples. These results are interpreted according to the hypotheses outlined above.

**Results:**

Genetic structure assessment allowed us to genetically differentiate most *Micromeria* species and supported their current classification. We found that populations on younger islands were significantly more genetically diverse and less differentiated than those on older islands. Moreover, we found that genetic distance on younger islands was in accordance with an isolation-by-distance pattern, while on the older islands this was not the case. We also found evidence of introgression among species and islands.

**Conclusions:**

These results are congruent with a scenario of multiple colonizations during the expansion onto new islands. Hybridization contributes to the grouping of multiple lineages into highly diverse populations. Thus, in our case, islands receive several colonization events from different sources, which are combined into sink populations. This mechanism is in accordance with the surfing syngameon hypothesis. Contrary to the surfing syngameon current form, our results may reflect a slightly different effect: hybridization might always be related to colonization within the archipelago as well, making initial genetic diversity to be high to begin with. Thus the emergence of new islands promotes multiple colonization events, contributing to the establishment of hybrid swarms that may enhance adaptive ability and radiation events. With time, population sizes grow and niches start to fill. Consequently, gene-flow is not as effective at maintaining the species syngameon, which allows genetic differentiation and reproductive isolation to be established between species. This process contributes to an even further decrease in gene-flow between species.

**Electronic supplementary material:**

The online version of this article (doi:10.1186/s12862-017-1031-y) contains supplementary material, which is available to authorized users.

## Background

Oceanic islands have always played an important role in evolutionary biology research [1]. The high availability of empty niches paired with the low migration rate from the mainland may contribute to a high prevalence for ecological speciation [1–3]. Hybridization might facilitate this adaptive evolution by increasing genetic diversity [4]. The processes linking hybridization and ecological adaptation as hypothesized by Seehausen [5], Seehausen et al. [4], and others (e.g. [6]), may be especially common on islands and form the basis for the prevalence of adaptive radiation on oceanic archipelagos. In agreement with this idea, Herben et al. [7] and Saunders and Gibson [8] suggested that multiple colonization events followed by hybridization occur in particular on archipelagos close to the mainland, promoting adaptive radiation due to the increase in genetic diversity. By comparing island taxa with their mainland relatives, it has been found that genetic variation in several insular populations was not significantly lower than their close relative in the mainland [9, 10]. In the case of a single colonization event, the founder effect would have caused significantly lower genetic diversity in island taxa. These observations led to the formulation of the surfing syngameon hypothesis where islands would constitute allelic sinks [11]. Through multiple colonization events originating from different sources, previously separated genotypes would be combined in hybrid populations (hybrid swarms), thus increasing genetic diversity. These populations might then differentiate ecologically into species that are still connected by gene-flow, thus forming a syngameon, i.e. a group of hybridizing species that evolve as one unit [11, 12]. Islands closer to the mainland are more likely to receive colonizers from the mainland, making them genetically more variable. The other islands are less likely to receive migrants from the mainland, but rather from members of the syngameon. This will lead to the loss of genetic diversity compared to the source due to the founder effect, and it may increase differentiation by way of the genetic dynamics of expanding populations due to allele surfing [11, 13].

The predictions of the surfing syngameon hypothesis are apparently in accordance with the distribution of genetic diversity across the Canary Islands, where genetic diversity is negatively related and differentiation positively related with island distance to the mainland when considering the overall genetic diversity per species per island [10]. The Canary Islands are a volcanic archipelago composed of seven islands located between 100 and 450 km off the Western Saharan coast. The islands originated from a westward moving hot spot, being older near the mainland and varying in age between 20.6 Ma (Furteventura) and 1.1 Ma (El Hierro). Like all volcanic islands, the Canaries present high levels of endemism as a consequence of their complex geomorphological composition and the high diversity of ecological zones [9, 14]. Each island is in a different stage of the oceanic island life cycle [15], some of them composed of older and younger parts [16]. For example, Tenerife resulted from the connection of three older palaeo-islands (Anaga, Teno, and Adeje) by a central volcano, and in Gran Canaria the southwest part of the island dates back to the Miocene (“palaeo-canaria”) and the northeast part to the Pliocene (“neocanaria”; [17]). This has been shown to have a high impact on the evolutionary history of species (i.e. [18–23]) that results in species occupying the younger part of the island being more genetically diverse but less differentiated among each other. This had been shown in our study system *Micromeria* (Lamiaceae) in Tenerife [22, 23].

Here, we expand our previous microsatellite study [23] to include all species of *Micromeria*, covering the whole Canarian archipelago, to investigate how inter-island colonization influenced the distribution of population diversity and pair-wise population differentiation throughout the archipelago. If multiple colonization and hybridization do not occur with high frequency, we would expect a decrease in genetic diversity from the older islands to the most recently colonized islands. Since the older eastern islands are expected to have been colonized first, they would be significantly more diverse than the younger western islands. In addition, because the likelihood of gene-flow from the mainland is lower in younger islands, genetic differentiation should be higher. In an alternative scenario, hybrid swarm creation would be more recent in younger islands, leading to a high diversity but higher homogeneity of populations, and genetic differentiation should decrease from the older to the younger islands. In the latter case the formation of the syngameon would have a higher significance during the diversification within the archipelago. Moreover, if single colonization events prevail, we do not expect to find gene-flow between species on different islands.

Here, these expectations are tested by investigating genetic structure, gene-flow among species on and between islands, and patterns of genetic diversity and differentiation of *Micromeria* on the Canary Islands. We interpret these results in light of the surfing syngameon hypothesis. The expectations outlined by Caujapé-Castells [11] are not completely met. We did find evidence of multiple colonization events providing high genetic diversity at the colonization front.

## Methods

### Biological system

**Fig. 1:**
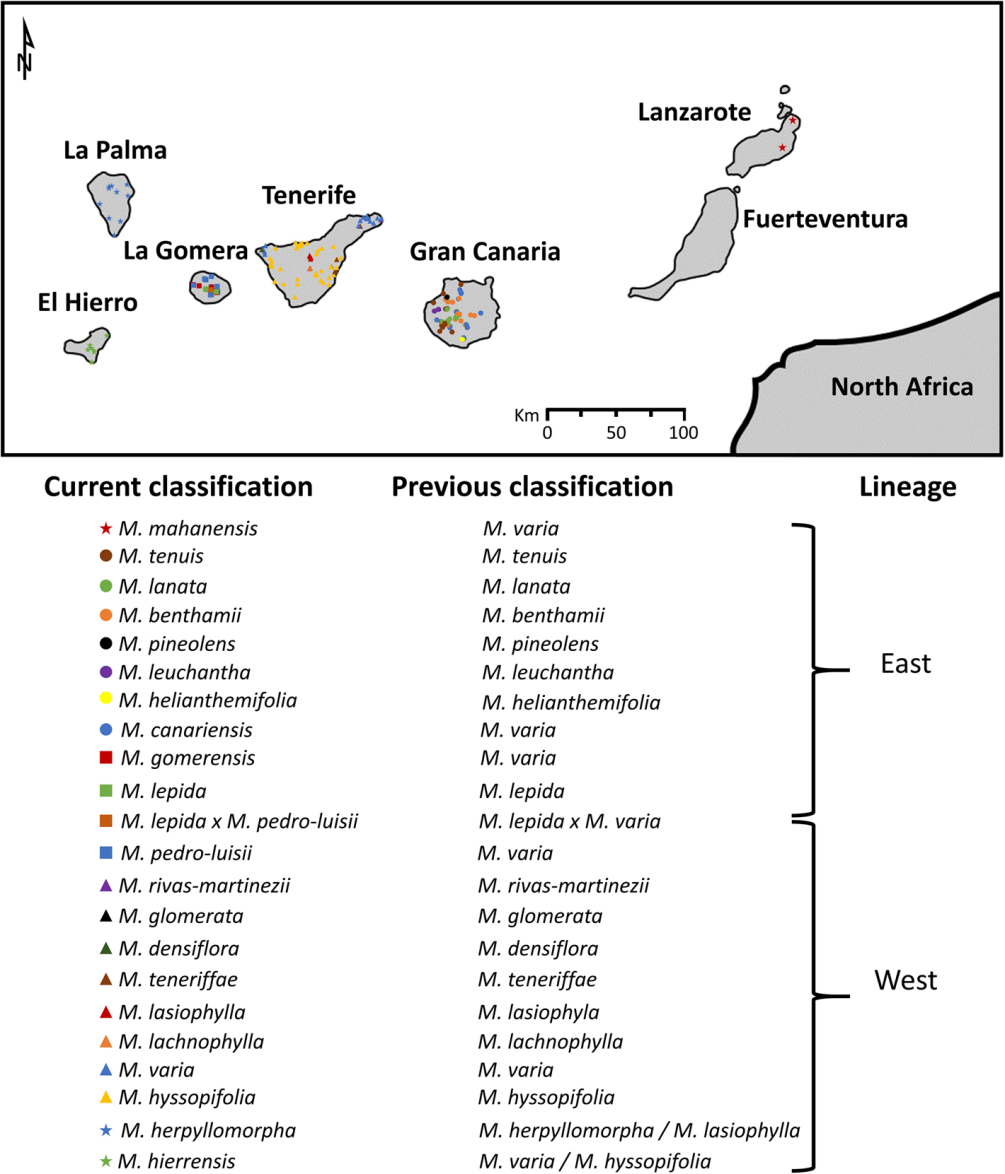
Map of the Canary Islands showing sampled localities and recognized species per island and group (East/West) to which they belong

*Micromeria* Benth. (Nepetoideae, Lamiaceae) is composed of shrubs, subshrubs and herbs with monoecious flowers pollinated by insects. Seed dispersal happens mostly by wind, but ants and water also contribute to this process [22, 24]. It is a monophyletic genus with ca. fifty four species [25] from which approximately 22 are present on the Canary Islands and Madeira [24, 26, 27]. They grow on all of the Canary Islands and in all ecological zones. As in many other Canarian taxa, molecular data show two main lineages of *Micromeria* on the Canary Islands archipelago [28, 29]. One includes the species found on the eastern islands of Gran Canaria, Lanzarote and Fuerteventura; the other contains taxa from the western islands of Tenerife, La Palma, and El Hierro (Fig. 1). Taxa from La Gomera belong to both lineages: *M. lepida* and *M. gomerensis* are part of the eastern lineage, while *M. pedro-luisii* is part of the western lineage [29].

*Micromeria* species from the Canary Islands were previously described by Pérez de Paz [24] based on morphological data and then reclassified by Puppo & Meimberg [26, 27] using phylogenetic information. Of all the species present on the Canary archipelago, six share similar morphological characters that lead to their previous classification as the same species (*M. varia*). These taxa occupy all islands except La Palma and have been recently separated based on molecular phylogenetic analyses [26, 27, 29] (Fig. 1).

Populations from the species previously classified as *M. varia* in Lanzarote and Gran Canaria as well as one of the subspecies from La Gomera form a monophyletic group, while the remaining ones are scattered in the phylogeny [29]. It was also shown that *M. varia* s.s. on Tenerife is genetically and morphologically more similar to *M. hyssopifolia* [22]. This variety of morphological features and their complex evolution allowed us to evaluate genetic variation at different levels: (i) by using only members of morphologically and genetically similar species, we replicated genetic patterns among individuals with independent phylogenetic positions but similar ecological and morphological features, which here is the case for a group composed of *M. hyssopifolia* and all species previously classified as *M. varia* (*M. varia s.l.*); (ii) by using only individuals from one lineage we compared the diversity among individuals belonging to the same monophyletic group; and (iii) by using the whole archipelago we assessed the diversity among all terminal branches of the *Micromeria* phylogeny.

Tenerife and Gran Canaria are the largest islands in the archipelago and contain the highest number of *Micromeria* species, eight and seven respectively. La Gomera presents three species and the remaining islands one species each. All *Micromeria* species are single island endemics. In Tenerife, it has been suggested that the composition of species of *Micromeria* is linked to the geological history of the island [22, 23, 29]. From the eight species occurring on this island, three have a narrow distribution restricted to the palaeo-islands: *M. densiflora* to Teno and *M. rivas-martinezii* and *M. glomerata* to Anaga. A fourth species, *M. teneriffae*, is also growing on the Anaga palaeo-island, but its range extends towards the southeast of the island up to Fasnia. Molecular studies suggest that these four species are older lineages that evolved before the connection of the three palaeo-islands by the formation of the central shield [22]. *Micromeria varia* s.s. presents a disjoint distribution growing on the palaeo-islands of Anaga and Teno, though molecular studies suggest it has a more recent lineage [22, 23]. The remaining three species are distributed on the central, younger part of Tenerife, *M. hyssopifolia* growing throughout the island from the coast up to the pine forest, *M. lachnophylla* from the pine forest up to the high desert, and *M. lasiophylla* in the Las Cañadas cliffs at the skirts of the Teide volcano. On Gran Canaria on the other hand, species distribution is apparently not correlated to the island’s evolution [29].

### Samples used and DNA isolation

A total of 945 individuals were included in this study, corresponding to all recognized taxa of *Micromeria* present on the Canary Islands (Fig. 1). These were collected during several excursions from 2010 to 2012 and some of these were already used in the studies of Curto et al. [30] and Puppo et al. [22, 23, 29, 31]. A total of 196 localities were sampled (Fig. 1). Each locality was considered to be an independent population and they were composed of 1 to 14 individuals (Additional file 1: Table S1).

In some of the analyses, individuals were put together into different groupings or levels: *island* (all individuals sampled in the same island); *species* (individuals from the same species according to Puppo & Meimberg [26, 27]); and *population* (all individuals belonging to the same population). More specifically, genetic diversity and pairwise genetic differentiation were estimated for *species and population* groups, whereas migration rates were calculated only between *island* and *species* groups.

DNA isolation was done using the Macherey-Nagel Plant DNA Extraction Kit (Macherey-Nagel, Düren, Germany) using 20 mg of dried leaf material according to Puppo et al. [23].

### Genotyping and marker quality control

The 16 microsatellite markers described in Puppo et al. [31] were amplified using the same multiplex primer combinations, fluorescent dyes, and PCR conditions from Puppo et al. [23, 31]. Genotyping was done in an ABI3130xl automatic sequencer (Applied Biosystems, Inc., Foster City, CA; USA) using an internal size standard (Genescan-500 LIZ; Applied Biosystems, Inc.). GeneMapper ver. 4.0 was used for allele scoring (Applied Biosystems, Inc.). A total of 96 individuals were genotyped two times to evaluate scoring consistency. After scoring, only markers showing data for a majority of the samples were used for further analysis. This led to the exclusion of one marker (6493). Puppo et al. [23, 31] did not find any significant deviation from the Hardy-Weinberg equilibrium, genotyping errors or a high amount of null alleles for any of the markers, so no further exclusions were necessary.

### Genetic structure assessment

Main genetic structure patterns were evaluated through distance and clustering analyses. These were used to show that the species defined by Puppo & Meimberg [26, 27] are independent units and that evidence of gene-flow between them is a consequence of introgression rather than a low degree of differentiation between them. Clustering analyses were conducted with the program STRUCTURE ver. 2.3.3 [32]. Considering that there are 22 species and that a structure within species can be found, the program was run between K = 1 and 30 for 500,000 generations after a burn-in period of 100,000 generations. For each K, 15 replicates were performed and the best value was obtained using the Delta K method as implemented in STRUCTURE Harvester [33]. All tests were conducted using the admixture model, since it is expected that individuals present multiple assignments to different clusters. The obtained results were summarized using the pipeline CLUMPAK [34]. We tested the statistical power of the best K value with the program POWSIM v. 4.1 [35]. This program tests for deviation from the null hypothesis of genetic homogeneity between simulated groups of samples. Population sizes were defined based on the divisions found in the STRUCTURE results, and the program ran for 1000 independent runs. Power is represented by the percentage of runs rejecting the null hypothesis. To look for further genetic structure signals within one island, six extra STRUCTURE analyses per island were performed. The analyses were conducted as described above, and the K values tested as well as a summary of the statistics of the STRUCTURE Harvester and CLUMPAK analyses can be found in Additional file 1: Table S2.

To assess the relationships among species, an UPGMA dendrogram was constructed using uNeiD distance [36] among *populations* in PAUP v. 4.0 [37]. Principal coordinates analyses (PCoA) were calculated in Genalex v. 6.41 (http://biology-assets.anu.edu.au/GenAlEx/) considering each population as a sample point and using three different datasets: 1) all populations, 2) Gran Canaria populations, and 3) populations assigned to the western group in the first analysis. Gran Canaria was tested independently because it was clearly separated from the other islands in the whole archipelago analysis. All analyses were calculated using pairwise genotypic distance among *populations* calculated in GenAlEx. We used these two analyses to explore the maximum amount of information from the data. While the PCoA provides information regarding how individuals may be grouped, the UPGMA dendrogram provides hierarchical information that can be used to assess lineage content.

### Gene-flow

Migration rates were calculated as proxy estimates of gene-flow. Evidence of gene-flow can be used to falsify a single colonization model and support the formation of species syngameon. Bidirectional contemporary and historical migration rates were calculated among species and island pairs using the programs BayesAss v3.0 [38] and Migrate [39], respectively. The combination of both provides a perspective of gene-flow patterns on two time scales. Because calculating migration-rates among all possible pairwise *population* combinations was not possible computationally, this was done using both *islands* and *species* as groups. In the event that a clear division within species was observed in the STRUCTURE analyses, these were considered to be different groups. In La Gomera species the two highly divergent lineages were treated separately in the island comparison. For Migrate, due to computational limitations, migration between islands was estimated using only *island* groups and migration contained on one island using *species* groups. In BayesAss, the output values correspond to the portion of individuals originating from the population that it is being compared to, while for the Migrate they correspond to the average number of migrants per generation. For BayesAss, only results with a minimum 95% confident interval value above 0.001 were considered to represent significant migration rates. In the case of Migrate, only migration rates significantly higher than 0 were considered. BayesAss parameters, such as mixing parameters, number of iterations, and burn-in were optimized in several runs. An optimal run was considered to have migration, allele frequency, and inbreeding coefficient acceptance rates below 0.6 and an effective sample size above 100 for all parameters (calculated with TRACER v1.5.0, [40]). The final analyses ran for 200,000,000 generations, excluding the first 100,000,000, and sampling every 1,000th generation for both groupings. For *species* pair analyses the optimal admixing values were 0.5 for both allele frequency and inbreeding coefficient, while for island pairs no adjustment was necessary. Migrate was run as described in Puppo et al. [23]. We used a burnin of 5,000,000 generations and estimates were sampled every 100th generation until a total of 50,000 were recorded.

### Genetic diversity and quantitative genetic differentiation

To test how genetic diversity and differentiation varied throughout the archipelago and if it was in accordance with the predictions from the surfing syngameon hypothesis, several statistics were calculated according to two categorical explanatory variables: *species* and *population*. We had a special focus on genetic diversity at the *population* level because it is not affected as much by sample size biases as the other groupings. Response variables for genetic diversity were assessed by calculating expected (H_E_) and observed heterozygosity (H_O_), allelic richness (Ar), and percentage of private alleles. Two private allele measures were calculated: one comparing each group (*population* or *species*) to the whole archipelago, the other comparing each group to the other groups from the same island. Response indices about genetic differentiation and quantification of genetic structure were calculated by estimating pairwise *F*_ST_ [41] and unbiased Nei’s distance (uNei) among groups. All measures were obtained using the same matrix but containing only populations with at least four individuals, a total 766 samples and 116 populations (Additional file 1: Table S1). Allelic richness was calculated with HP-Rare [42], while the remaining measures were estimated with GenAlEx 6.41 (http://biology-assets.anu.edu.au/GenAlEx/)

These measures on the *species* and *population* levels were used to perform statistical analyses for evaluating the differences in genetic diversity and differentiation among the three explanatory variables (island, species and age). The clear division of the Canary Islands by their age resulted in two age levels, namely young island species and old island species. Tenerife is a special case because formerly independent islands became later connected, and the so called palaeo-islands have had a more pronounced botanical singularity than the other islands. The species were grouped according to their position in the phylogenetic analysis to account for the possibility of recent range shift between old and young substrates. Thus the group of young island species were composed of species from the central area, *M. lachnophylla, M. lasiophylla* and *M. hyssopifolia,* and including the closely related species *M. varia* s.s., which expanded onto the palaeo-islands from the central part (see *Biological system*). When species were grouped according to their current main distributions, the results only changed slightly but were still significant. After the calculation of pairwise genetic differentiation, a third category was considered for this measure: differentiation between old and young areas. In summary, two age levels were defined for the genetic diversity measures (old and young) and three levels for the genetic differentiation measures (among old, among young, between old and young).

Genetic differentiation results may reflect differences among lineages rather than age. This was overcome by doing analyses on genetic diversity and differentiation including only members of the western lineage (data subsets “within west”) that occupy both old and young islands. Age-related biases were assessed by performing a test including only the recently diverged taxa from the *M. varia* /*M.hyssopifolia* species complex.

Regarding statistical analysis, data exploration was done following Zuur et al. [43] for all datasets (all observations, *M. varia* and *M. hyssopifolia* on western islands). According to histograms the response variables (H_O_, H_E_, Ar, *F*_ST_, uNeiD) appeared to be normally distributed. Variance homogeneity of the responses among the levels of categories at the species level (island, age) and at the population level (island, age, species) was not given in all cases according to Fligner Killeen Tests. Further, we found out that the explanatory variables (island, age, species) were highly related (i.e. collinear) to each other. Therefore it was not possible to include them in the same models (e.g Generalized Linear Model), because this would lead to incorrect parameter estimation [43]. As a consequence we decided to analyze the differences of genetic diversity and differentiation with non-parametric Kruskal-Wallis tests to maintain analytical consistency.

Differences in genetic diversity on a *species* level were assessed for island and age while at the *population* level these differences were analyzed among island, age and species. However, in the data subset of *M. varia* and *M. hyssopifolia,* differences in genetic diversity were only assessed in terms of island age because of small sample size due to data aggregation. The same statistical framework was applied to genetic differentiation, but, at the *species* level, species was also included as an explanatory variable. We had to deal with an increased number of single tests for each response variable because the Kruskal Wallis Test can only account for differences in response variables among a single factor (equal to non-parametric one-way ANOVA). Therefore, we corrected the initial significance level (α = 0.05) using the Sidak [44, 45] method given by (Formula 1),

1 $$ {\alpha}^{\prime }=1-{\left(1-\alpha \right)}^{\frac{1}{k}} $$

where k is the number of test applied to a given response variable. Because 14 single tests were used for diversity response variables and 18 single tests were performed for differentiation responses, we adjusted the α Level to 0.003. All statistical tests described in the paragraph above were done in R v. 3.3.2 [46].

### Isolation by distance

We performed an isolation by distance test (IBD) to evaluate if, alongside island age, the effect of geographical distance is another factor explaining genetic differentiation. To do this we compared populations pairwise *F*_ST_ with geographical distance in kilometers using the Isolation By Distance Web Service v. 3.23 [47]. We ran the program for 1000 replications for both log-transformed and non-log-transformed data.

## Results

### Genetic structure

**Fig. 2:**
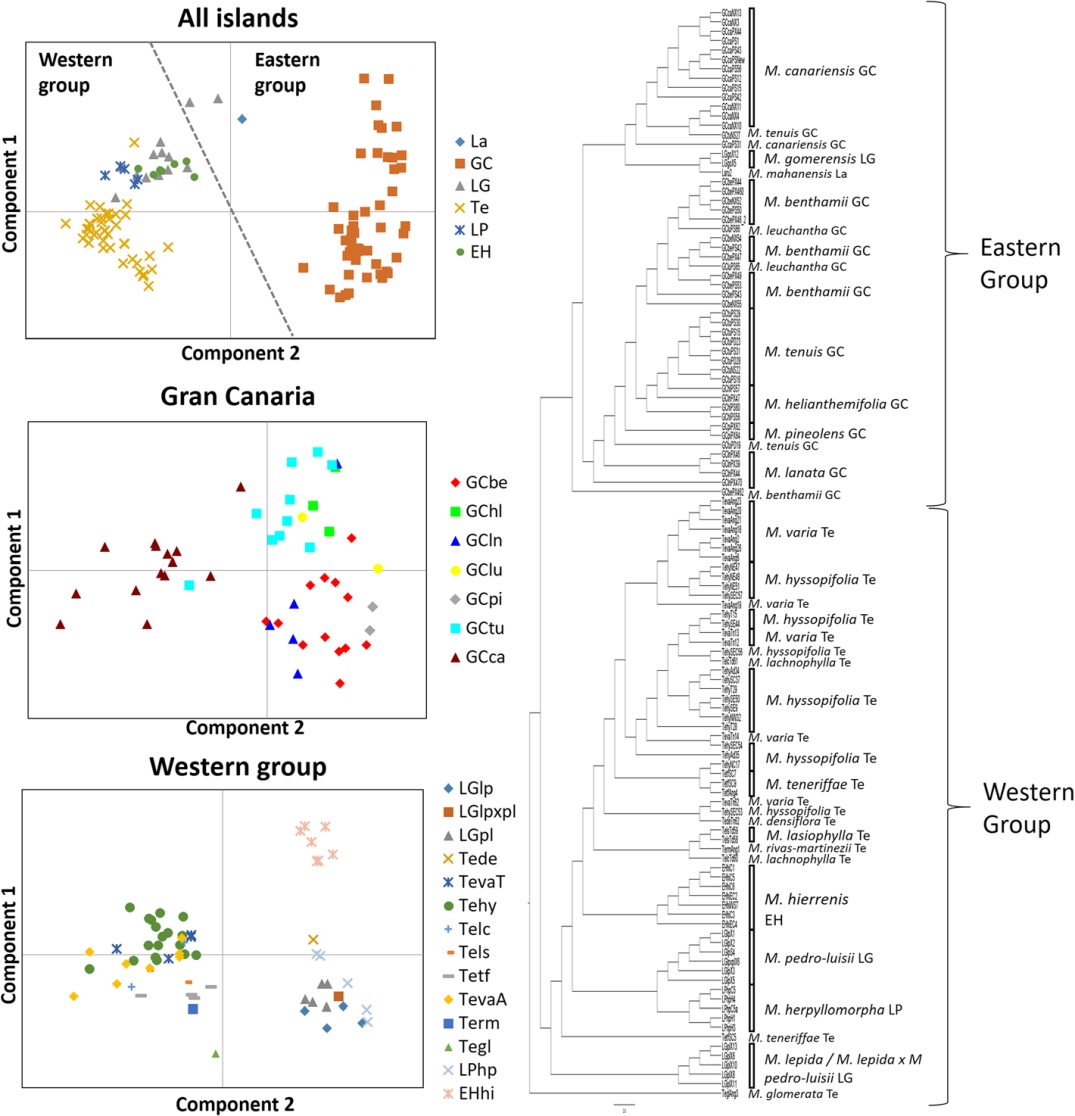
Principal coordinates analyses (PCoA) and UPGMA dendrogram using pairwise uNeiD distances between all populations with at least four individuals. For the PCoA, three analyses were performed: all populations; populations belonging to Gran Canaria; and the ones belonging to the western islands and *M. lepida* from La Gomera. In the analysis containing all populations a dashed line divides the eastern and western lineages. The vertical and horizontal axes explained respectively: 33.1 and 8.3% of the variation for the all island analyses, 31.9 and 10.4% for the Gran Canaria analyses and 17.6 and 13.9% for the western group analyses. Islands are represented by the following abbreviations: La (Lanzarote). GC (Gran Canaria). LG (La Gomera); LP (La Palma); EH (El Hierro)

Patterns of genetic distances (uNei among populations) were visualized using both dendrograms and PCoA analyses (Fig. 2). They were generally congruent with previous phylogenetic and similarity analyses [22, 23, 29]. Populations tend to cluster into two main groups, generally corresponding to the division between eastern and western islands. Populations from La Gomera were assigned to both groups: *M. gomerensis* with the eastern islands; and *M. lepida* and *M. pedro-luisii* within the western islands). Genetic structure was congruent with species delimitation for both analyses. Interestingly, one *M. teneriffae* population (tetSC5) clusters within the other western islands, which may indicate gene-flow among these islands.

**Fig. 3:**
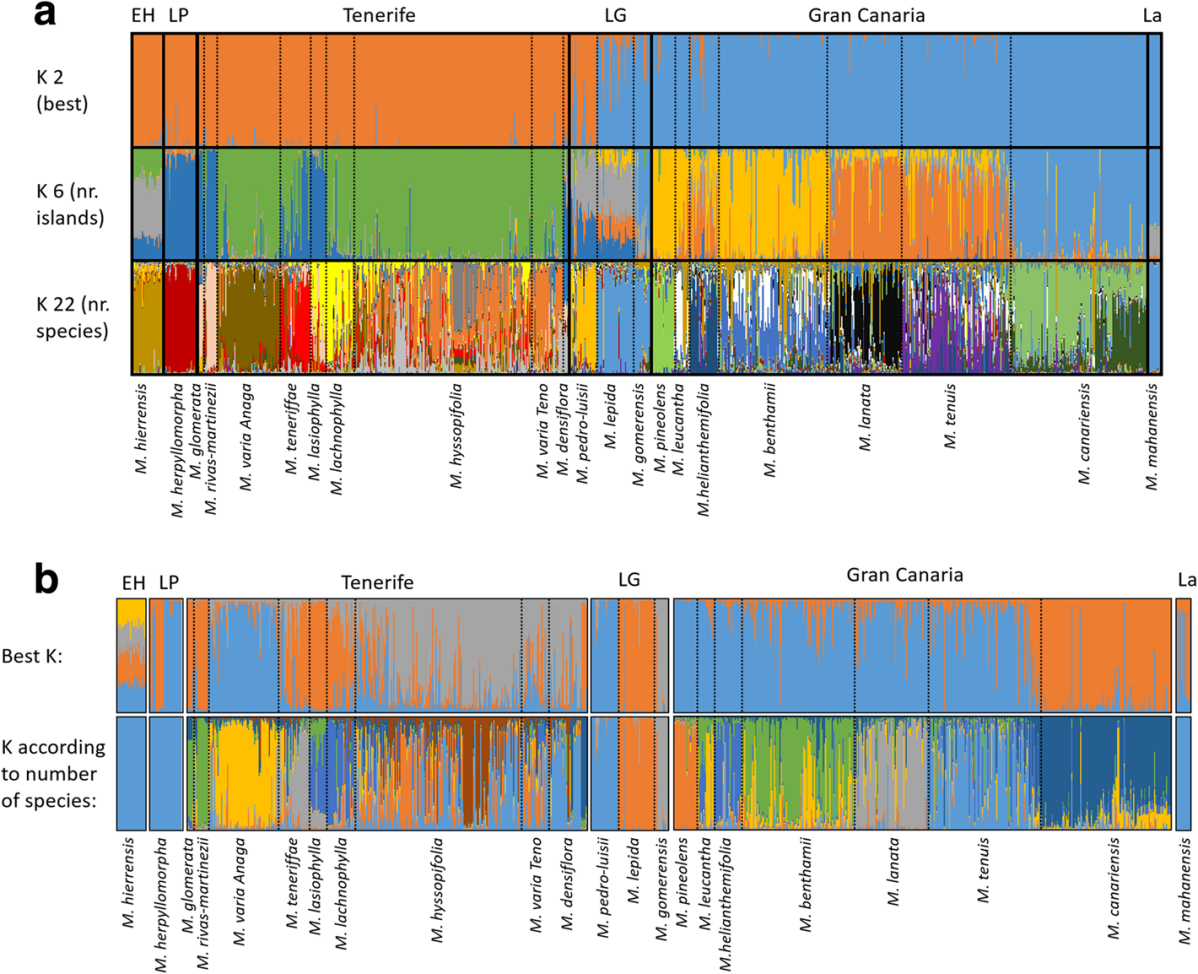
Bayesian clustering analyses with STRUCTURE. **a** Corresponds to the analyses of the complete archipelago for the best K (2), and for K according to the number of islands (6) and species (22). **b** Corresponds to individual analyses per island where the upper part shows the analyses for the best K values and the bottom one to K value corresponding to number of species per island. The K values per island can be found in the Additional file 1: Table S2

In the STRUCTURE analysis considering the complete archipelago (Fig. 3a), the optimal K value according to Evanno’s method was two (Additional file 2: Figure S1). To evaluate clustering further, we show larger K values corresponding to the number of islands (K = 6) and number of species (K = 22). The LnProb mean and similarity scores after combining the 15 runs in CLUMPAK are summarized for all K values in Additional file 1: Table S2. The statistical strength of the runs increased with the K value (Additional file 1: Table S2); however, in none of the analyses did the majority of power runs reject the null hypothesis of homogeneity, indicating that our data is not powerful enough to accurately divide all the samples into the K number of groups. Thus, any ambiguous assignment may reflect this limitation and not biological processes such as introgression. Nevertheless, some conclusions can be made. The division in K = 2 (best K) corresponds to the division between the eastern and western islands. In K = 6, some further divisions can be found within these lineages. In K = 22, with almost all islands, most species were separated into individual clusters with the exception of two species groups: 1) *M. gomerensis* and *M. lepida* from La Gomera, together with *M. mahanensis* from Lanzarote; and 2) *M. glomerata* and *M. rivas-martinezii* from Tenerife. When a STRUCTURE analysis was conducted with each island independently, the only improvement was found for La Gomera, where three species (*M. gomerensis*, *M. lepida* and *M. pedro-luisii*) are separated (Fig. 3b). The power of the analysis was also low (Additional file 1: Table S2).

### Gene flow

**Fig. 4:**
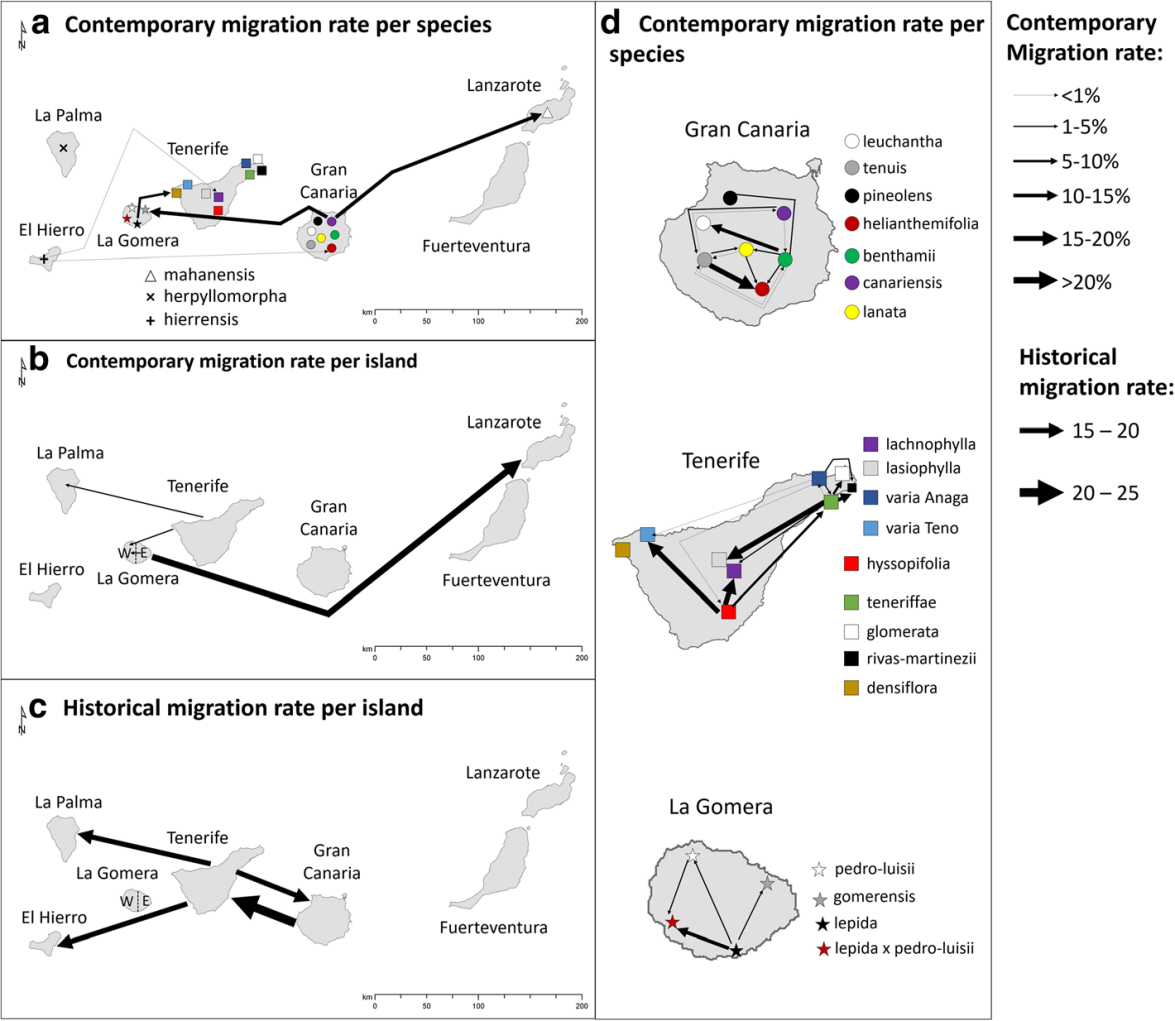
Representation of recent and historical migration rates calculated with BayesAss and Migrate, respectively. All arrows correspond to migration rates with 95% confidence intervals above 0.001 for BayesAss and 0 for Migrate. Arrow thickness is proportional to the migration rate. Panels **a** and **b** correspond to contemporary migration rates calculated between islands using species and islands as groups, respectively. Panel **c** shows historical migration rates between islands groups calculated with Migrate. These were the only significant values obtained from this program. In Panel **d**. contemporary migration rates between species within islands are shown

Recent migration rates calculated with BayesAss varied between 0.07 and 24.44% among *island* groups and between 0.18 and 19.9% among *species* groups (Additional file 1: Table S3). Past migration rates calculated with Migrate varied from 2.8 to 32.3 individuals per generation among *island* groups and from 3.38 to 32.77 among *species* groups (Additional file 1: Table S4). These were only significant among *island* groups. Figure 4 shows all significant migration rates from both analyses.

Recent connectivity among islands was mostly found within the eastern and western lineages (Additional file 1: Table S4; Fig. 4). This corresponded to gene-flow among the islands of Lanzarote, Gran Canaria and La Gomera in the east, and among the islands of Tenerife, La Gomera and La Palma in the west. Additionally, recent connectivity among the following lineages were found: among species of La Gomera, from El Hierro to Gran Canaria, and from La Gomera to Tenerife. Past migration, inferred through Migrate, was found from Tenerife to Gran Canaria, La Palma, and El Hierro; and from Gran Canaria to Tenerife.

Only recent migration was significant within islands connecting 12 out of 56 species pairs in Tenerife and 14 out of 42 species pairs in Gran Canaria (Additional file 1: Table S4; Fig. 4). Although Gran Canaria showed more connections, the intra-specific migration rate in Tenerife was higher, with an average of 7.39% compared to the 3.53% for Gran Canaria. Within Tenerife, *M. hyssopifolia* and *M. teneriffae* were the source of interspecific migration, *M. hyssopifolia* to the central species, *M. teneriffae* to the palaeo-endemic species. In Gran Canaria, all widespread species functioned as a source of migration. On La Gomera, the *M. lepida x pedro-luisii* hybrid received migration from both potential parental species. Additionally, *M. lepida* was the source of migration for the other two species on La Gomera.

### Genetic diversity and differentiation

**Fig. 5:**
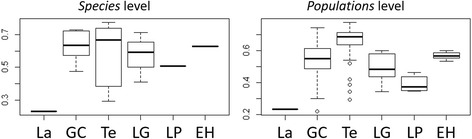
Box plots of expected heterozygosity (H_E_) per island calculated considering *species* and *population* groups

Genetic diversity and differentiation was estimated as heterozygosity, uNeiD, *F*_ST_, percentage of private alleles, and allelic richness (Additional file 1: Tables S4 and S5). At the *species* level, taxa from Lanzarote was the least diverse. *Micromeria hyssopifolia* presented the highest H_E_ (0.77), *M. varia* from Teno the highest H_O_ (0.77), and *M. helianthemifolia* the highest Ar (8.9). Per *population*, H_O_ varied between 0.14 and 0.68, H_E_ between 0.22 and 0.78, and Ar between 1.91 and 14.33. Overall, genetic diversity seemed higher in the western islands, the island of Tenerife being the most diverse (Fig. 5).

At the *species* level, differentiation was consistently the lowest among *M. varia* s.s. from Teno and *M. hyssopifolia* (*F*_ST_ = 0.02, uNei = 0.14) and was consistently the highest between Lanzarote and *M. glomerata* (*F*_ST_ = 0.62; uNei = 3.09). At the *population* level, pairwise *F*_ST_ ranged from 0.05 to 0.66 and uNei from 0.13 to 5.16.

**Fig. 6:**
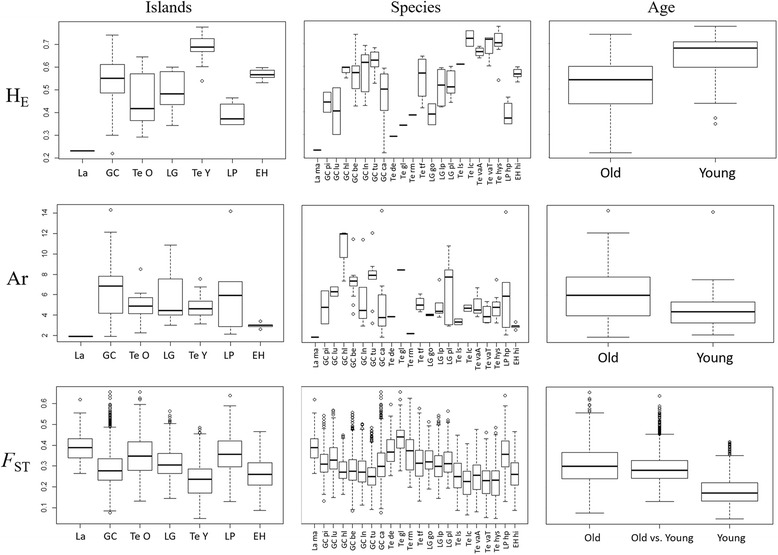
Representation of expected heterozygosity (H_E_) and *F*_ST_ per population used as proxy of genetic diversity and differentiation. For each measure there is a graph per island per species and per age class. Here only the test including all samples is shown. Graphs for the other tests are shown in Additional file 2: Figure S2

Except for H_O_ in the western lineage, heterozygosity measures were significantly higher in younger islands than in older ones, which also corresponded to significant differences between island and species (Table 1; Fig. 6; and Additional file 2: Figure S2). These differences were also significant considering the measures calculated per *species* for the complete dataset. Overall, as well as on *population* and *species* levels, heterozygosity decreased in the direction of east to west (Fig. 6). Allelic richness per *populations* was significantly higher on older islands for the complete dataset, while no differences were found in the other tests. Differences among islands and species were also significant for Ar, being more pronounced at the *population* level than at the *species* level.

**Table 1:** Results of Kruskal Wallis analyses of island age over genetic diversity for three datasets: whole archipelago, *M. hyssopifolia* and taxa belonging to the *M. varia* complex; and taxa belonging to the western lineage (Tenerife, La Palma and El Hierro and *M. pedro-luisii* from La Gomera)

Dataset	Level	Test	H_O_			H_E_			Ar		
			DF	Chi^2^	*P*-value	DF	Chi^2^	*P*-value	DF	Chi^2^	*P*-value
All samples	Populations	Islands	6	50.5	<.0001	6	65.81	<.0001	6	24.89	0.0003
		Species	21	71.51	<.0001	21	84.66	<.0001	21	53.33	0.0001
		old/new	1	24.19	<.0001	1	30.96	<.0001	1	10.35	0.001
	Species	Islands	6	14.27	0.02	6	12.69	0.04	6	6.28	0
		old/new	1	7.36	0.006	1	4.62	0.03	1	0.15	0.69
*M. varia* and *M. hyssopifolia*	Populations	Islands	4	29.48	<.0001	4	43.02	<.0001	4	16.71	0.002
		Species	7	33.81	<.0001	7	46.05	<.0001	7	18	0.01
		old/new	1	20.59	<.0001	1	33.73	<.0001	1	0.05	0.8
	Species	old/new	1	4.08	0.04	1	4.08	0.04	1	2.08	0.1
Within West	Populations	Islands	5	31.43	<.0001	5	40.04	<.0001	5	14.22	0.01
		Species	11	33.08	<.0005	11	46.16	<.0001	11	22.35	0.02
		old/new	1	6.57	0.01	1	13.86	0.0001	1	1.49	0.2
	Species	Islands	4	9.19	0.06	4	9.18	0.05	4	0.98	0.9
		old/new	1	4.81	0.02	1	3.49	0.06	1	0.006	0.9

Significance was adjusted to α ≤ 0.003 by the Sidak method due to an increased number of tests per response

**Fig. 7:**
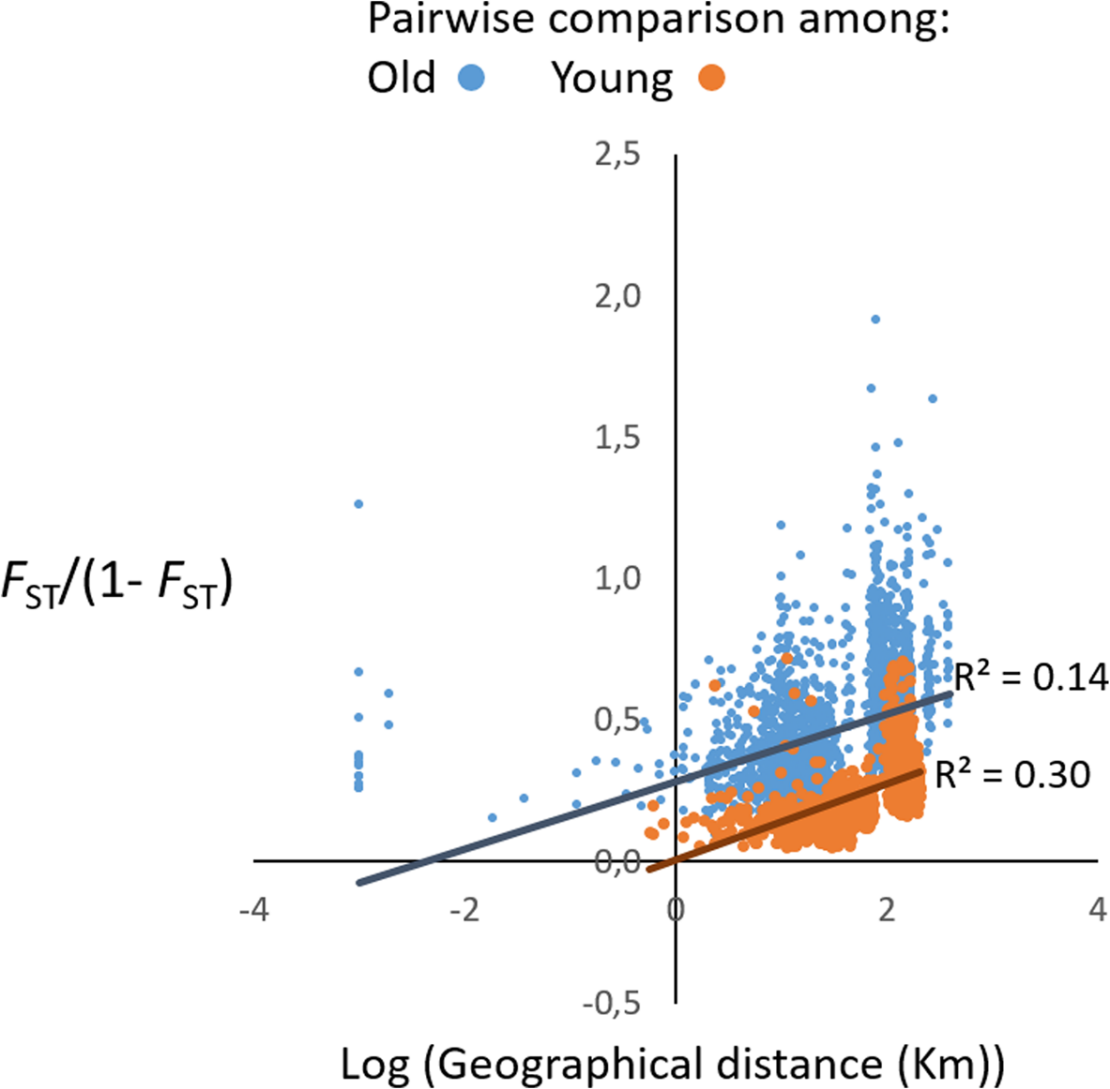
Plot of pairwise *F*_ST_/(1- *F*_ST_) with log of geographical distance in kilometers. The points in *Blue* correspond to pairwise comparisons among populations in older regions and the points in *Red* to comparisons in younger regions. *Blue* regression line and upper R^2^ correspond to pairwise comparisons between young populations, orange regression line and lower R^2^ correspond to pairwise comparisons between old populations

Differentiation among *populations* and *species* was significantly higher on older islands (Table 2, Fig. 6, and Additional file 2: Figure S2). This difference can be seen in further detail when genetic differentiation was plotted in comparison with geographical distance (Fig. 7). Populations in older regions are always more differentiated, independently of the geographical distance between populations. The patterns found for the comparisons among age classes reflected how genetic differentiation varied across the archipelago. While for the western islands dataset there was a gradual decrease of genetic differentiation from older to younger islands, for the other two datasets this pattern was only evident for *F*_ST_ (Fig. 6).

**Table 2:** Results of Kruskal Wallis analyses of island age over genetic differentiation for three datasets: whole archipelago,; *M. hyssopifolia* and taxa belonging to the *M. varia* complex; and taxa belonging to the western lineage (Tenerife. La Palma and El Hierro and *M. pedro-luisii* from La Gomera)

Dataset	Level	Test	*F*_ST_			uNeiD		
			DF	Chi^2^	*P*-value	DF	Chi^2^	*P*-value
All samples	Populations	Islands	5	1366	<.0001	5	76.38	<.0001
		Species	21	2991	<.0001	21	572.6	<.0001
		old/new	2	2734	<.0001	2	4138.7	<.0001
	Species	Islands	5	33.6	<.0001	5	11.27	0.04
		Species	21	185.6	<.0001	21	64.88	<.0001
		old/new	2	102.4	<.0001	2	76.42	<.0001
*M. varia* and *M. hyssopifolia*	Populations	Islands	4	831.17	<.0001	4	252.9	<.0001
		Species	7	857.3	<.0001	7	282.6	<.0001
		old/new	2	2105	<.0001	2	1934.3	<.0001
	Species	Islands	4	18.91	0.0008	4	5.78	0.22
		Species	7	21.46	0.003	7	8.18	0.32
		old/new	2	30.66	<.0001	2	25.70	<.0001
Within West	Populations	Islands	3	592.4	<.0001	3	339.46	<.0001
		Species	11	1204	<.0001	11	651.61	<.0001
		old/new	2	1209	<.0001	2	927.42	<.0001
	Species	Islands	3	0.80	0.8	3	6.96	0.07
		Species	11	55.92	<.0001	11	46.89	<.0001
		old/new	2	68.85	<.0001	2	61.95	<.0001

Significance was adjusted to α ≤ 0.003 by the Sidak method due to an increased number of tests per response

The portion of private alleles per *species* varied from zero for several taxa to 8.5% for *M. hyssopifolia*. When compared to the remaining species from the same island, *M. pedro-luisii* had the highest portion of private alleles (51.85%). In contrast *M. rivas-martinezii* shared all alleles with other Tenerife species. At the *population* level, the portion of private alleles varied from 0% for several populations of multiple species to 6.45% for one population from La Palma (*M. herpyllomorpha*). This population also presented the highest portion of private alleles from a single island (62.06%). Private alleles were not as informative when assessing the variation among the defined categories. Nevertheless, the number of private alleles per island was higher in younger islands when the dataset containing the whole archipelago was considered.

### Isolation by distance

Geographical distance was weakly correlated with *F*_ST_ (*r* = 0.36; R^2^ = 0.13). When the analyses were made using either only populations from older regions or only populations from younger ones, it was possible to see that isolation by distance (IBD) was much more pronounced in younger regions (*r* = 0.63, R^2^ = 0.39) than in older ones (*r* = 0.45; R^2^ = 0.20). IBD better explained divergence for when just these two subsets of data were considered, rather than all individuals.

## Discussion

### Evidence of gene-flow between islands

We found that gene-flow through hybridization does not only exist on individual islands, but that it also seems to link different islands to one another. This is an important prerequisite that indicates that connectivity between islands might be higher than generally expected, creating a subsequent impact on colonization ability and diversification. With other types of markers, direct detection of gene-flow between islands was impossible (data not shown), mostly because of limited sampling and a lack of differentiation between taxa.

Using the analyses of different programs, we found evidence for contemporary and historical gene-flow between islands. This was indicated by recent connectivity within the eastern and western lineages and by historical connectivity among the central species of Tenerife, Gran Canaria, and La Gomera. High historical migration rates are found between the two major lineages (east and west), but recent gene-flow between them is found to be sparse. Barriers to gene-flow might accumulate with time and can affect how genetic diversity is distributed. Nevertheless, introgression between these lineages is still found, especially through hybridization in La Gomera. This indicates that although highly diverged, these lineages are not yet completely isolated, which can be a consequence of continuous genetic exchange during the divergence of these lineages [7].

### Factors shaping gene-flow within islands

In our previous study, we detected gene-flow between species within Tenerife, most pronouncedly between the species in the central part of Tenerife and members of the *M. varia* group. We showed here that this is not only the case with Tenerife, but also on all islands where more than one species is found. Interestingly, Tenerife showed a larger amount of gene-flow than Gran Canaria when the total amount of migration is considered. Both islands are composed of old and young substrates from the Miocene and Pleistocene [17, 48]. Puppo et al. [22, 23] found that genetic structure among species correlated with these regions in Tenerife, which was confirmed by our data. Differences in gene-flow among species, and age estimates of phylogenetic relationships between the two largest islands, might be related to differences in the geological formation of the islands. While both are of similar maximum age, Tenerife as we know it today was created by the unification of three older islands by a central area, and Gran Canaria got its current shape by subsequent additions of land in the northeast direction [17, 48]. These different island formation processes resulted in different ways of land occupation. While the central, younger part of Tenerife was occupied by colonization, younger areas of Gran Canaria were occupied by the expansion of pre-existing populations. In the next section, we hypothesize that hybridization may be common during the colonization process.

### Genetic differentiation, diversity patterns, and the surfing syngameon hypothesis

The current model of the species syngameon postulates that, within the archipelago, genetic diversity decreases with colonization steps. This is mostly a consequence of the islands closer to the mainland being more likely to work as allelic sink than the remaining ones. Our data supports rather a scenario where the younger islands receive a significant amount of genetic diversity, in which differentiation is lower than expected when considering the surfing syngameon hypothesis for the Canary Islands. The existence of gene-flow within and between islands may explain this pattern, where high levels of admixture increase genetic diversity and can ultimately have an adaptive effect. Here, we see that both distance to the source of colonization and time have an effect on diversity.

Within populations, genetic diversity was higher in younger islands, and genetic differentiation was always higher for older islands. Additionally, we observed a weak isolation by distance pattern (IBD), which may be a consequence of a stepwise colonization process from the oldest islands in the east to the younger islands in the west. Our data is composed of several species, and if they were completely reproductively isolated from each other, the differentiation pattern would be better explained by species segregation events that are not necessarily correlated with geographical distance. The fact that the IBD pattern is more pronounced in younger regions indicates that these species are genetically more homogeneous and more connected to gene-flow. This connectivity ultimately results in a species syngameon composed of highly genetically diverse and minimally differentiated units. For some tests, allelic richness results either did not differ between old and young islands, or showed a contradictory pattern when compared to the heterozygosity levels. This measure only considers the number of alleles per population [49]. Thus, the observed pattern, for example, in older islands (high Ar and low H_E_ and H_O_) is a consequence of high number of alleles with low frequency in the populations. This indicates that these populations were previously highly diverse, but the frequency of many of their alleles decreased probably through drift, which is more pronounced in populations less connected through gene-flow.

Our results might be explained by the following mechanism: on younger islands the colonization process is still ongoing, so they contain a low number of taxa that may not have yet occupied all niches available, so they display a lower number of populations and individuals. Here, colonization may have a higher impact on the genepool of already established populations. On older islands, taxa had time to expand and are now represented by higher number of populations, individuals, and species. Thus, gene-flow onto these islands might have a lower effect and does not contribute much to changes in the genepool of established populations. Therefore, colonizers should have a lower effect on preventing population differentiation on the older islands, as well as a lower chance of increasing within-population diversity. This mechanism is similar to the one described by the niche-preemption hypothesis [50, 51] where competition prevents the establishment of new colonizers. The main difference is the fact that multiple colonization events continue to exist, however with time they just lose their ability to affect the genetic pool of the islands. Consequently, the decreasing impact of gene-flow promotes differentiation among populations, which contributes to a higher reproductive isolation and increases the pace of differentiation through a snowball effect.

### The range expansion mechanism of the species syngameon

Considering a source-sink dynamics, the surfing syngameon hypothesis implies that the sink populations can have a cumulative effect on genetic diversity by maintaining their connection to their source through gene-flow. We can apply these assumptions to explain our results by presenting a scenario of inter-island colonization with continuous gene-flow. After the emergence of an island, individuals that can hybridize will colonize it from different sources. In this case the syngameon expands with the appearance of new islands and prevents the loss of genetic diversity at the colonization front by buffering founder effect. In the Canary Islands this expansion moves in an east-west direction. As the syngameon expands, populations on old islands might become isolated, speciate, and thus become disconnected with increasing reproductive isolation. This dynamic is similar to the model of species range shifts, in which new populations in the expansion front have a high connectivity to the source, and thus they receive higher amounts of gene-flow. Although differentiation between populations is low, the rear relict populations become increasingly isolated, showing low differentiation within populations and high differentiation between populations [52]. In *Micromeria*, the syngameon is therefore not only expanding and shifting, but it is continuously being recreated at the colonization edge, which might thereby increase the potential for ecological speciation at the expansion front. A recent update of the surfing syngameon hypothesis incorporated the role of island ontogeny on shaping the species syngameon [53] and the present work with *Micromeria* may lead to further refinements of this hypothesis.

### Taxonomic implications

We chose to use microsatellites, which are mostly used at the intraspecific level. Nevertheless, these also have successfully been used to evaluate species boundaries and test hypotheses related to the species concept [54, 55], supporting our approach.

The genetic structure found in the present study was mostly congruent with previous phylogenetic studies. All species described in Tenerife had already been supported with microsatellite data [23]. In the present study, we were able to do the same for most of the remaining *Micromeria* species, supporting the last *Micromeria* species delimitation from Puppo & Meimberg [26, 27]. STRUCTURE was able to define unique clusters for most species. With the exception of *M. gomerensis* and *M. mahanensis,* and *M. glomerata* and *M. rivas-martinezii*, all remaining species were differentiated in the analyses containing only individuals from the eastern and western groups. These species were differentiated in the other analyses (PCoA and UPGMA dendrogram) and in previous studies using nuclear markers [29], so this pattern may be a consequence of characteristics intrinsic to the microsatellites. These markers are mostly neutral [56], making them more susceptible to introgression [57]. Thus, the high levels of introgression found in this system may have resulted in lower genetic structure when microsatellites were used.

### Future directions

Further tests of the hypotheses outlined here would benefit from a denser sampling of the eastern island of Lanzarote and the inclusion of samples from Fuerteventura. These are the oldest islands of the archipelago, and thus according to our expectations should be the least diverse and the more genetically differentiated. A higher sampling could result in an increase in genetic diversity, since it would be more likely to sample more alleles. However, given the results in other, older regions that were densely sampled, we do not expect this would be significant enough to change our observations. The island of Fuerteventura was connected to Lanzarote at several times in the past [15]. In fact, what appears today as two islands is still only one volcanic building, where erosion has caused its middle part to be submerged, thus giving the appearance of two different islands. In an earlier study [28], the *Micromeria* individuals from Fuerteventura were closely related to the ones from Lanzarote, indicating that they may constitute previously contiguous populations and thus should show similar genetic patterns. Besides sharing the same geological history, both islands present similar geomorphological features. They are mostly shaped by erosion that contributes to the loss of most of their previous altitudinal range, which resulted in a high extinction rate [3] and thus the loss of genetic diversity.

In this study we decided to focus on the extension of the surfing syngameon predictions to colonization between islands within an archipelago. However, it would be interesting to measure the amount of gene-flow from the mainland and its influence on the genetic diversity of the archipelago taxa. At the moment it is still not known which mainland species are the closest relatives to the Canary Islands. The last phylogeny produced with mainland species showed that both Iberian and Moroccan species are the main candidates, but the analyses were not powerful enough to decide which one was more closely related to the Canary Islands species [58]. We are currently working on expanding our sampling in order to estimate the amount of gene-flow from the mainland.

### Conclusions and outlook

The results of our study of neutral genetic variation for *Micromeria* across the Canarian archipelago indicate the existence of a syngameon that may facilitate colonization and speciation on oceanic islands. The species syngameon seems to expand and shift in accordance with a range expansion scenario. Island colonization has many parallelisms with other evolutionary processes, and theoretically the model proposed here could be applied to scenarios where a range expansion is accompanied by adaptive speciation. We showed how genetic diversity is affected by gene-flow and hybridization, but we do not assess its adaptive implications. To do so, genomic approaches where both coding and non-coding variation is compared with ecological and morphological features might successfully be implemented.

## Additional files

Additional file 1: Table S1. List of all localities with information regarding the number of individuals sampled, species classification according to Puppo and Meimberg [26, 27], and coordinates. **Table S2.** Summary statistic of the STRUCTURE analyses performed. This includes the K values tested and which ones were the best. It also includes the mean LnProb and similarity score of the 15 replicates obtained with CLUMPAK. Lastly we also include the results of the power analysis done with POWSIM, presented as percentage of runs that contradicted the null hypothesis of homogeneity of the data. **Table S3.** Migration rates calculated with BayesAss. Values correspond to percentage of individuals from the species (3.1) or island (3.2) in the upper line originated from species (3.1) or island (3.2) in the left column. The second value corresponds to the 95% confidence interval. Migration rates significantly higher than zero are marked in bold. **Table S4.** Migration rate calculated with Migrate. Values correspond to average number of individuals migrating per generation. Source species or islands are in the left column and sink species or islands in the upper line. Values with 95% intervals above zero (under brackets) are marked as bold. (XLSX 35 kb)

Additional file 2: Figure S1. Structure Harvester results for mean likelihood (above) and DeltaK values (below) per K. A) Analysis for the complete archipelago was conducted with 15 replicates of K = 2 to K = 30, resulting in the best K of 2, which is marked by the arrow. B) STRUCTURE analyses per island were conducted with different values of K (see Additional file 1: Table S2). The best K values are also marked with an arrow. **Figure S2.** Representation of expected heterozygosity (H_E_) and *F*_ST_ per population used as proxy of genetic diversity and differentiation. For each measure there is a graph summarizing it per island, per species, and per age class. From top to bottom the box-plots correspond to the different datasets used, including: all samples, only *M. varia* and *M. hyssopifolia*, and Western lineage (Tenerife, EH, LP, and part of LG). (DOCX 487 kb)
