# Supplementary material for: Genetic diversity and differentiation patterns in Micromeria from the Canary Islands are congruent with multiple colonization dynamics and the establishment of species syngameons
Source: BMC Evol Biol. 2017 Aug 22;17:198. doi: 10.1186/s12862-017-1031-y (PMC5568322; doi:10.1186/s12862-017-1031-y)

Additional file 2


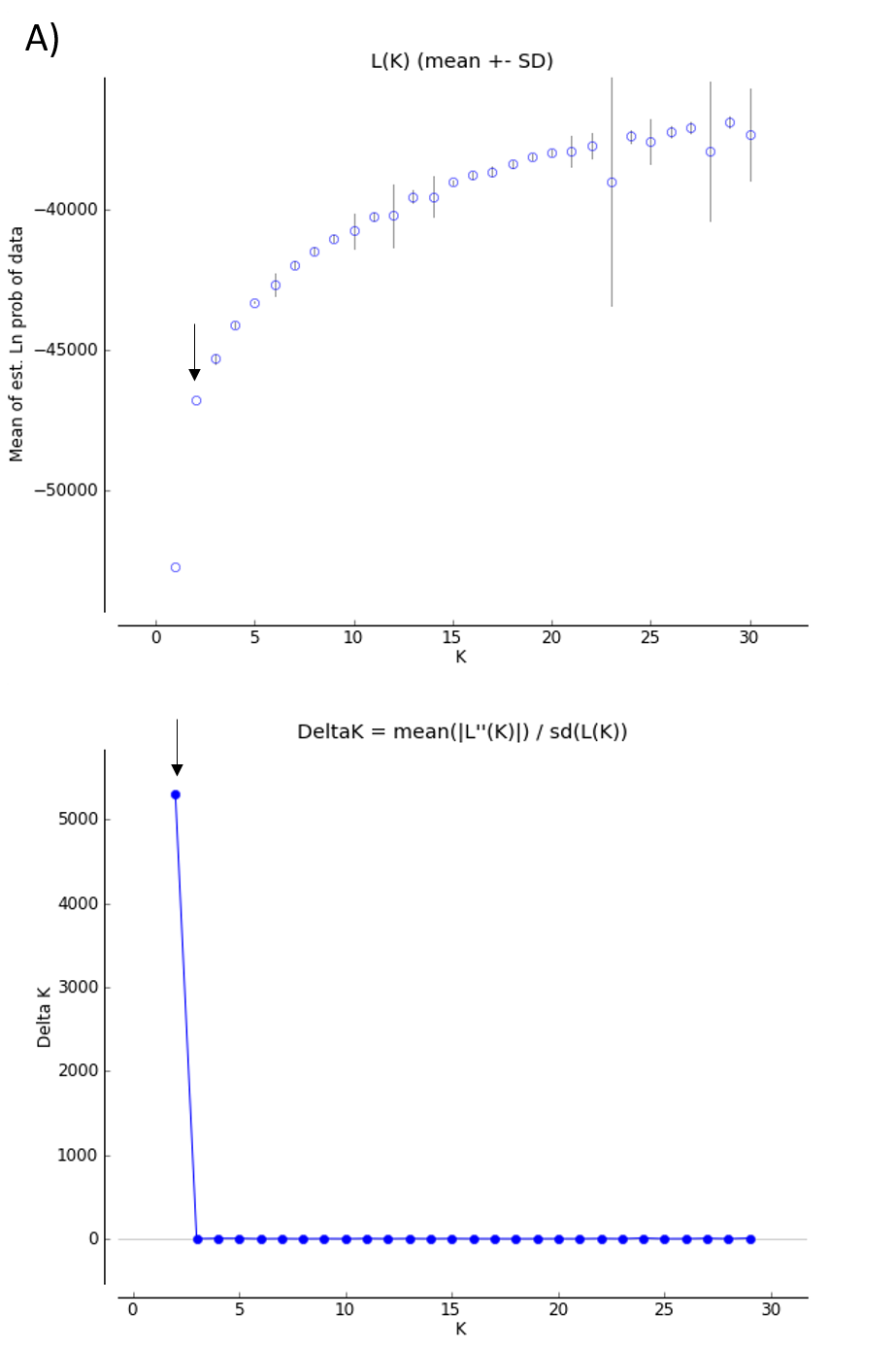
Figure S1. Structure Harvester results for mean likelihood (above) and DeltaK values (below) per K. A) Analysis for the complete archipelago was conducted with 15 replicates of K=2 to K=30, resulting in the best K of 242, which is marked by the arrow. B) STRUCTURE analyses per island were conducted with different values of K (see supplementary Table S2). The best K values are also marked with an arrow.

Figure S1. Continue


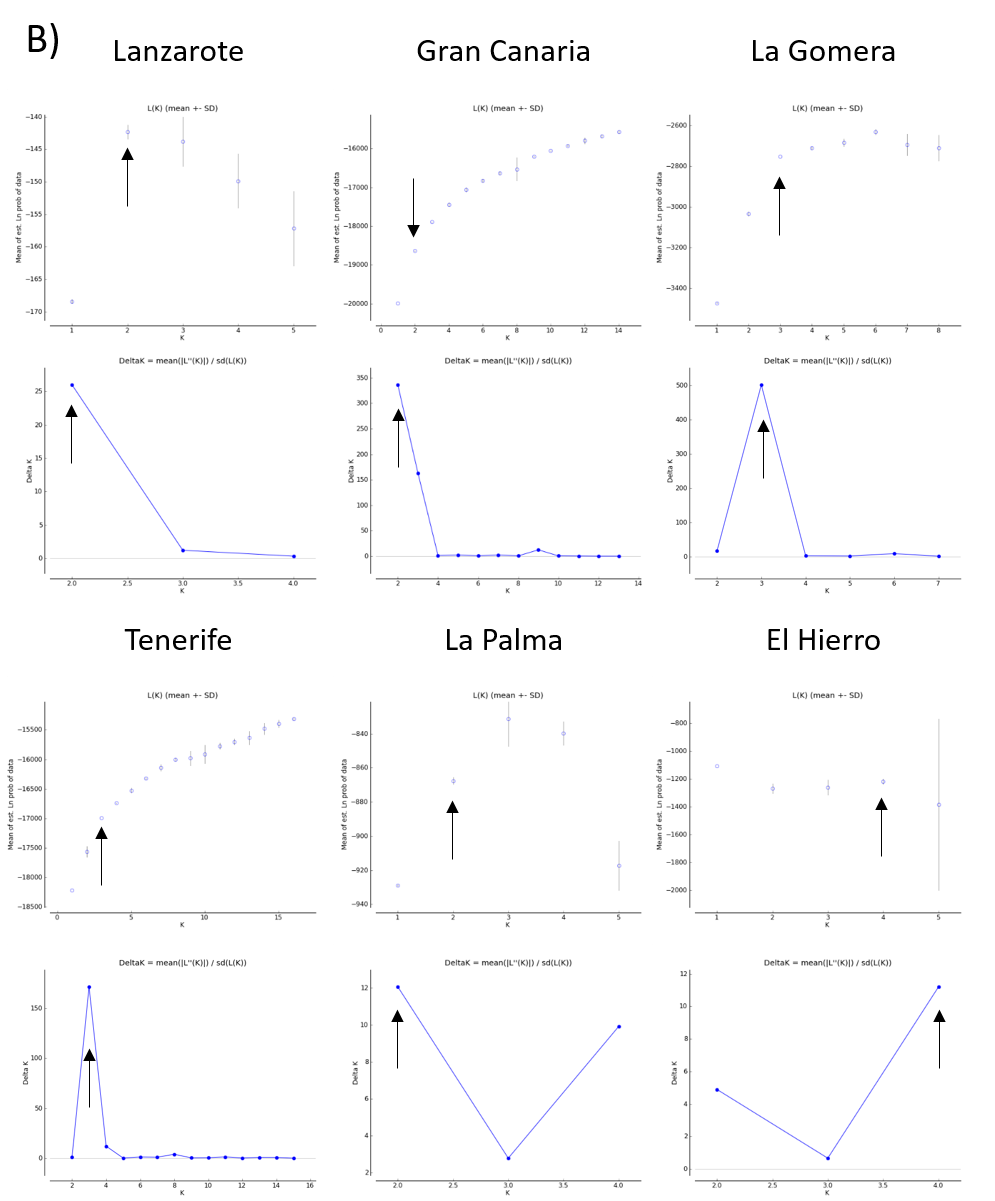


Figure S2. Representation of expected heterozygosity (HE) and FST per population used as proxy of genetic diversity and differentiation. For each measure there is a graph summarizing it per island, per species, and per age class. From top to bottom the box-plots correspond to the different datasets used:, including: all samples, .only M. varia and M. hyssopifolia, and Western lineage (Tenerife, EH, LP, and part of LG).


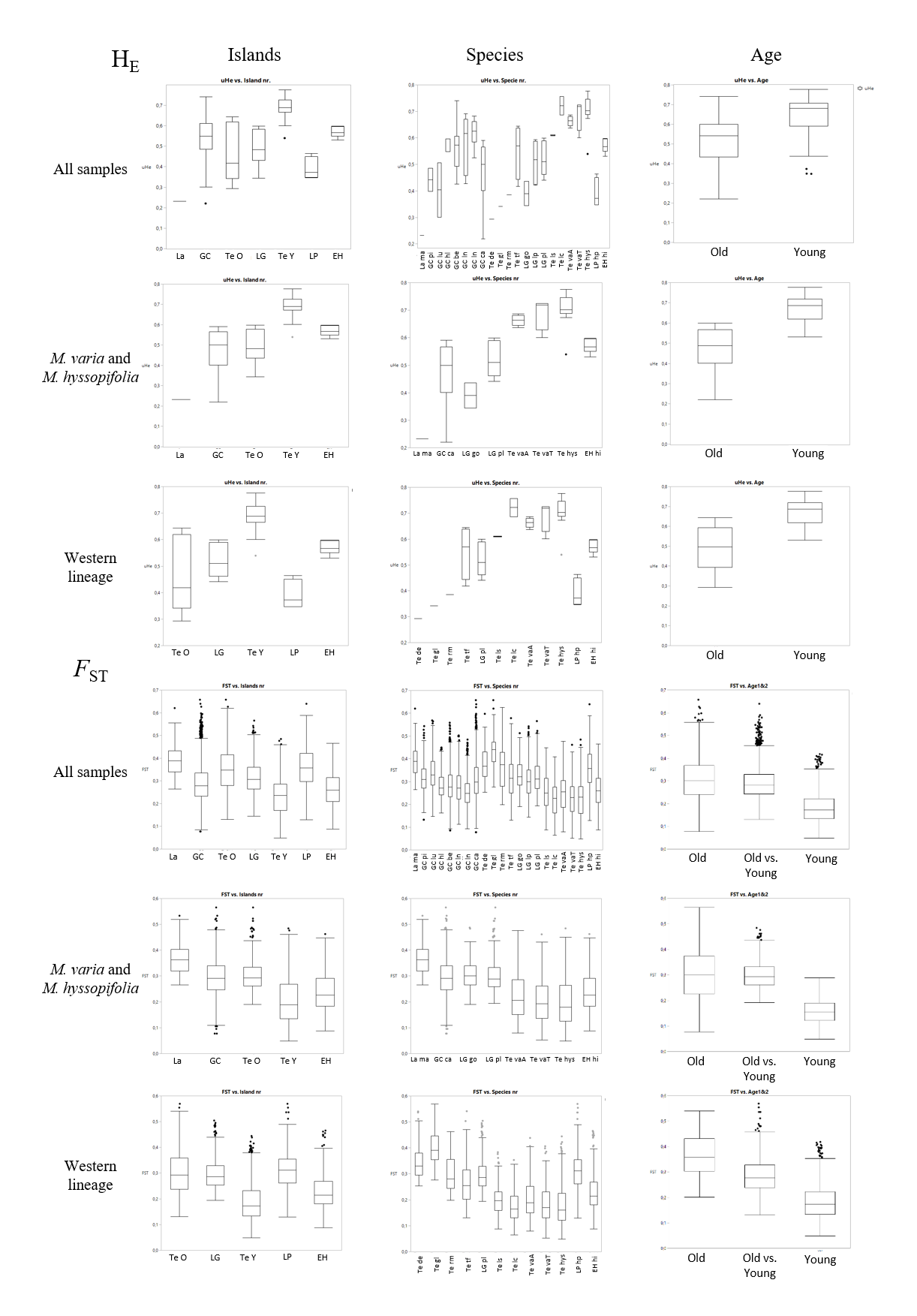

Supplement: Supplementary file 2 — Structure Harvester results for mean likelihood (above) and DeltaK values (below) per K. A) Analysis for the complete archipelago was conducted with 15 replicates of K = 2 to K = 30, resulting in the best K of 2, which is marked by the arrow. B) STRUCTURE analyses per island were conducted with different values of K (see Additional file 1: Table S2). The best K values are also marked with an arrow. Figure S2. Representation of expected heterozygosity (HE) and F ST per population used as proxy of genetic diversity and differentiation. For each measure there is a graph summarizing it per island, per species, and per age class. From top to bottom the box-plots correspond to the different datasets used, including: all samples, only M. varia and M. hyssopifolia, and Western lineage (Tenerife, EH, LP, and part of LG). (DOCX 487 kb) [file 12862_2017_1031_MOESM2_ESM.docx]
